# Fendrix^®^ Vaccine Effectiveness in Healthcare Workers Who Are Non-Responsive to Engerix B^®^ Vaccination

**DOI:** 10.3390/vaccines9030279

**Published:** 2021-03-19

**Authors:** Juan José Tejada-Pérez, Juan José Vázquez-Vicente, María Renée Herrera-Burgos, Francisco Gabriel Martín-Martín, Tesifón Parrón-Carreño, Raquel Alarcón-Rodríguez

**Affiliations:** 1 Faculty of Health Sciences, University of Almería, Carr. Sacramento, s/n, 04120 La Cañada, Almeria, Spain; tpc468@ual.es (T.P.-C.); ralarcon@ual.es (R.A.-R.); 2 Risk Prevention Service, Poniente Hospital Entrepreneurial Public Health Agency, Ctra Almerimar, 31, 04700 El Ejido, Almeria, Spain; juanjose.vazquez@ephpo.es (J.J.V.-V.); marenee_dc@hotmail.com (M.R.H.-B.); franspreven@gmail.com (F.G.M.-M.)

**Keywords:** Hepatitis B, vaccination, fendrix, healthcare workers, occupational health

## Abstract

Hepatitis B (HBV) is a pathogen virus with transmission mechanisms that include contact with the infected blood or bodily fluids of the infected organism. Nowadays, healthcare workers are one of the most exposed groups to HBV. Conventionally, completing a vaccine series dosage with Engerix B^®^ lowers this risk by providing workers with immunity to the virus. However, through the years, we have encountered nonresponsive health personnel to the Engerix B^®^ vaccine; hence, the Occupational Health Service of Poniente Hospital studied the Fendrix^®^ adjuvanted vaccine as an alternative vaccine to develop immunological responses in healthcare workers who do not respond to vaccination with Engerix B^®^. In our study, we employed a vaccination schedule with the Fendrix^®^ vaccine, performing serology tests on the cases after the application of each dose. The results obtained showed humoral immunity in 92.3% of the cases, with a remarkable increase in antibody titer after the first doses. These encouraging results support the future inclusion of this vaccine as one possible alternative for the immunization to HBV for healthcare workers nonresponsive to Engerix B^®^.

## 1. Introduction

Vaccines are the primary prevention tool against various biological agents through the development of humoral immunity. This humoral immunity, or immunological memory, is achieved by exposing an individual to the pathogen by one of the following mechanisms: direct exposure, by the inoculation of a less virulent strain of the microbe (attenuated vaccine) or indirect exposure, by the inoculation of antigenic fragments of the pathogen, which are normally expressed in the organism during the regular infection process (inactivated vaccine) [1].

Although both types of vaccination have a high rate of efficiency, inactivated hepatitis B vaccine has been found to have a lower immunization rate following complete dosage guidelines than other vaccines routinely administered to the general population and health personnel. In this way, although many risk factors are considered for a vaccine to be effective or not, there is still much to discover and specify [2], but in the meantime, we must try to develop immunity in all people who have not yet generated immune protection through the usual vaccines.

The Spanish National Healthcare System provides a wide vaccination coverage which employs both attenuated and inactivated vaccines. The annual vaccination schedule is targeted at the at-risk population groups: children and the elderly [3,4].

The Spanish Healthcare System also recommends the vaccination of other at-risk population groups, either due to previous pathologies such as immunosuppression, asplenia, and/or transplanted patients [5,6], or due to a high exposure risk, such as essential healthcare workers [7].

In a previous function performed by our research group, the efficiency and importance of the correct vaccination of healthcare workers has been studied, since this group is constantly exposed to multiple biological risks [8]. In said study, the high efficiency of vaccines administered against measles, rubella, mumps, chickenpox, and hepatitis B in nonimmunized health personnel was proven. However, the vaccine administered against the hepatitis B virus (HBV) presents the lowest efficiency of the set—84.8% of postvaccination immunity when correctly following the vaccination series schedule [8,9].

The Engerix B^®^ vaccine, regularly used for HBV immunization, consists of a 1 mL solution of an HBV surface pathogen (standard dose 20 mcg/mL). The schedule for this vaccine consists of three doses: an initial dose, a second dose after 1 month, and a third dose after 6 months [10,11]. If the postvaccination serology test performed at the seventh month after the first dose results in a reading of anti-HBs < 10 mIU/mL, a new second full dose schedule is proposed to be repeated, and if a low titer of anti-HBs is observed, the worker is considered nonresponsive and no further vaccination procedure is performed.

The adjuvanted vaccine Fendrix^®^ is of restricted use, with a high immunological capacity, and its target population are renal insufficiency patients. Acting through the antigen release system, this vaccine can increase the availability of antigen in antigen-presenting cells. A delay in antigenic clearance and an increase in antigen response is achieved in specific physiological location (through aluminum salts), as well as through the use of immune-enhancers such as 3-O-desacyl-4-monophosphoryl lipid A (MPL), which directly activate cell receptors and induce the release of cytokines [12].

Currently, this vaccine is not included in any vaccination program for health personnel; however, the Occupational Health Service of Poniente Hospital was authorized by the hospital pharmacy for its application for healthcare workers nonresponsive to Engerix B^®^. The recommended number of doses is 4: an initial dose and after 1, 2 and 6 months after receiving the vaccination [13,14].

The objective of this study is to demonstrate the high effectiveness of the Fendrix^®^ vaccine, and to provide the required evidence for its future inclusion in the vaccination plans of the National Health Authorities in Spain for healthcare workers.

## 2. Material and Methods

### 2.1. Study Description

A prospective study to determine the effectiveness of the Fendrix^®^ vaccine was performed on healthcare providers nonresponsive to Engerix B^®^.

### 2.2. Participants

In total, 26 healthcare workers were selected by the Occupational Health Service of Poniente Hospital in southeastern Spain (Almeria, Spain) in the 2011–2020 period. None of the initially recruited workers abandoned the study.

The inclusion criterion for all health personnel was that after the application of 2 complete vaccination schedules with Engerix B^®^, they did not develop immunity against HBV, objectively demonstrated by the necessary serology tests (to detect possible antibodies against HBV). The exclusion criteria included those healthcare workers with prior HBV immunity or those without immunity but that had not complied with the Engerix B^®^ vaccination stipulations. In this study, only one group was assigned (there is not a control group). There was no randomization or masking.

All participants were informed about the study and signed the appropriate informed consent form.

### 2.3. Engerix B^®^

Engerix B^®^ is the vaccine of choice to develop immunity against HBV. The vaccine is comprised of a surface antigen of the viral shell at a concentration of 20 μg/mL in a solution containing sodium chloride, sodium phosphate and water, preserved at a temperature between 2 and 8 °C.

The doses applied to the hospital healthcare workers prior to their inclusion in this research were those recommended by the Spanish Agency of Medicines and Medical Devices (AEMPS), with inoculation at months 0, 1 and 6 [10].

### 2.4. Fendrix^®^

Fendrix^®^ is an adjuvanted vaccine with higher immunogenic capacity compared to other traditionally employed vaccines against Hepatitis B. This vaccine was to be used only in patients with renal insufficiency, helping them develop better protection against HBV. For this, in addition to the surface antigen, Fendrix^®^ contains two compounds the adjuvant molecule MPL in conjunction with the surface antigen of HBV dissolved in aluminum phosphate. The recommended preservation temperature range is between 2 and 8 °C.

The doses applied also followed the indications by the AEMPS, with inoculation at months 0, 1, 2, and 6 [13].

### 2.5. Procedure

Healthcare providers who did not develop immunity to HBV after completing the 3-dose vaccination schedule with Engerix B^®^ at months 0, 1, and 6 (anti-HBs level < 10 after the vaccination) were informed about the possibility to develop immunity against Hepatitis B by commencing a vaccination schedule with Fendrix^®^.

Participants were informed about the procedure being outside of the recommendation by the AEMPS and about the possible contraindications and adverse effects of the Fendrix^®^ vaccine.

**Scheme 1:**
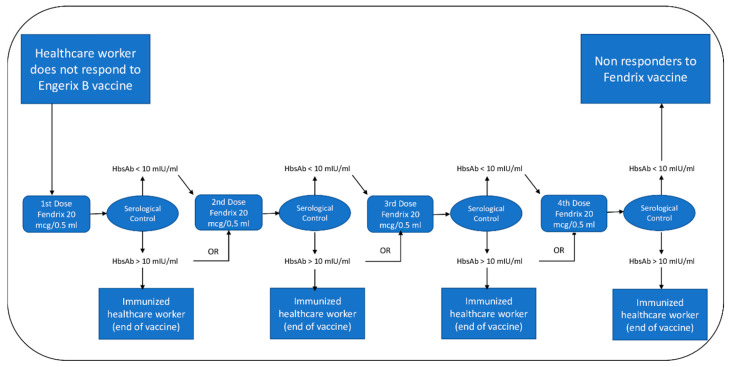
Fendrix^®^ vaccine vaccination protocol.

Those health personnel who consented to participate in this study were vaccinated following the previously mentioned indications, with a vaccination schedule consisting of four doses applied at months 0, 1, 2 and 6. Thirty days after administering each dose, a serology test was performed with the objective of determining at which point each individual developed immunity against HBV. Due to the high cost of Fendrix^®^, after demonstrably achieving immunity against hepatitis B, each participant was given the option to not complete the original vaccination schedule. For those participants who after the completion of the full vaccination schedule the result of the serology test was nonconclusive, an additional dose of Fendrix^®^ was offered together with additional serology testing. (Scheme 1).

### 2.6. Statistical Analysis

A database was created with the variables studied in this article and statistical data analysis of said data was performed using IBM SPSS version 22.0, 2013 [15].

First, a descriptive analysis of continuous variables was performed, expressed as averages and standard deviations. Categorial values were expressed alongside their respective frequencies and percentages. Afterwards, in order to determine whether the continuous variables fit a normal distribution or not, a Kolmogorov–Smirnov test was performed. For the comparison of the variables following a normal distribution, Student’s *t*-distribution was employed, whereas non-normally distributed variables were compared with the Mann–Whitney U test. In the bivariate analysis of qualitative variables, the chi-squared test (χ^2^) was employed with *p* < 0.05.

### 2.7. Ethical Considerations

The approval for the application of the Fendrix^®^ vaccine outside of the common recommended usage by the AEMPS was provided by the hospital pharmacy, with the requirement of informing the participants of the study about possible contraindications and adverse effects. With this approach, signing an informed consent form was required prior to the participation in this study for the application of the Fendrix^®^ vaccine.

This study was performed following Good Clinical Practice Guidelines, and it conformed to the Declaration of Helsinki, observing all ethical requirements for medical research on human beings. This study also followed the principles and conventions of the Council of Europe related to human rights and biomedicine, and all stipulations present in the Spanish law regarding bioethics.

Patient voluntary consent was conditio sine qua non in all cases, and all participants could retract their consent at any time and thus abandon the study.

Each worker was assigned an ID to provide them with anonymity. This study was approved by the provincial ethics and research committee number 12 on 18 December 2019 (research code: PI_19_41).

## 3. Results

**Figure 1:**
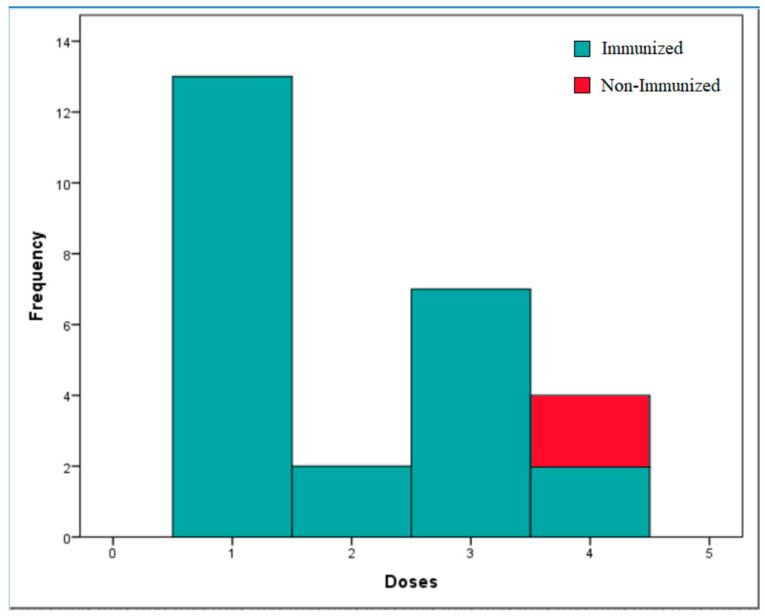
Immune status according to the doses of Fendrix^®^ administered. This histogram shows the immune response of health personnel according to the doses of Fendrix^®^ vaccine inoculated (*n* = 26).

A total of 26 healthcare workers participated in the study. Participant average age was 46.12 ± 8.07 years, and 57.7% were women. Regarding dosing, 50% of the workers were inoculated with one dose (*n* = 13), 7.7% were inoculated with two doses (*n* = 2), 26.9% were inoculated with three doses (*n* = 7), and the remaining 15.4% were inoculated with four doses (*n* = 4) (Figure 1). The average antibody titer obtained from the participants was anti-HBs = 325 ± 304.9 mIU/mL, thus achieving immunity in 92.3% of healthcare workers (*n* = 24).

Statistically significant differences were found for the development of immunity against the hepatitis B virus depending on the number of doses employed and the antibody titer levels produced by the participants (anti-HBs). In this respect, the average number of doses required for immunity development was found to be 1.92 ± 1.1, with an average anti-HB level of 351.83 ± 302.14 mIU/mL. The 7.7% of healthcare providers who did not develop immunity received four doses and presented an anti-HBs titer of 3.1 ± 4.38 mIU/mL (Table 1).

Furthermore, statistically significant differences were found depending on the age of the vaccinated workers. The average age of workers who developed immunity was 45.58 ± 8.19 years, whereas the average age of workers who did not develop immunity was 52.5 ± 0.71 years (Table 1).

No statistically significant differences between the gender of the participants and the rest of the analyzed variables—immunity development, age, number of doses, and antibody titer—were found (Table 2).

Neither could a statistically significant correlation between the antibody titer and the number of doses inoculated be found, or with the age of the healthcare workers. However, in the case of the antibody titer and the number of doses given, a weak, negative correlation was found (*p* = 0.083) (Table 2).

## 4. Discussion

The present study is part of an ambitious larger project which advocates the extension of the prescriptive usage of the Fendrix^®^ vaccine in health personnel nonresponsive to Engerix B^®^ vaccination. It must be mentioned that this group deals with high work-related biological risks. The type of possible accidents with biological risk include stabbing with sharp medical instrumentation and contact with potentially contaminated bodily fluids, amongst others. These work-related risks are prevalent and typically underreported [16], hence the exposure risk to hepatitis B contagion by contact with contaminated sources is potentially elevated, giving rise to situations in which appropriate chemoprophylaxis procedures cannot be undertaken [17]. Thus, providing healthcare providers with immunity against HBV is of the utmost importance. It must also be noted that HBV is the pathogen with the highest seroconversion capacity among the microbiological work-related biological risk accidents, with a seroconversion capacity of 20–30% in exposure cases in its positive E antigen (HBeAc+) replicative form [18]. By increasing the personnel with immunity against HBV, the negative effects of hepatitis B on healthcare workers could be prevented in most cases [19], alongside the reduction in the direct and indirect costs on the National Healthcare System resulting from newly infected workers [20].

Internationally, several studies endorse the inclusion of the Fendrix^®^ vaccine outside its common indications and its inclusion in the immunization schedule to develop immunity in people nonresponsive to the currently used HBV vaccines. These studies have found an efficacy of the Fendrix^®^ vaccine in over 85% of the patients, results similar to those found in this research [21,22].

In Spain, a research study similar to this one can be found in the literature that sought to demonstrate the efficacy of the adjuvanted vaccine and support its inclusion in the vaccine calendar of nonresponsive health personnel [23]. In said article, the same population group was targeted for the testing of the Fendrix^®^ vaccine, with similar vaccination schedules and conditions to our own. This investigation demonstrated high efficacy (>90%) and high efficiency, with serological immunization in over 50% of healthcare workers after the inoculation with the first dose. In our project, these results have been complemented with the description of their prevalence, and alongside this we have established a relevant relation between the number of Fendrix^®^ doses of inoculation and immunity development. This can be due to the elevated response potential adjuvanted vaccines cause, which favor greater local inflammatory reaction and activating the immunological response faster [24].

A significant correlation between the number of generated antibodies and the number of doses given cannot be demonstrated with our data. This correlation has, however, been found in other studies in which Fendrix^®^ was used, with up to a 9-fold increase in antibody count compared to a complete Engerix B^®^ vaccination schedule [25,26]. One possible reason our investigation could not determine this correlation might be the number of cases evaluated. Increasing the population studied, either by the inclusion of new participants within our health center or from patients coming from other health facilities in a future project, might allow us to find a definitive correlation between these two variables. If this were the result, it would further help the case for the correlation between the cause of greater inflammatory reactions to adjuvant vaccines and the greater-scale immunological response [26].

On another note, this study demonstrates that vaccine efficacy is higher in younger patients, with decreased immunity development in patients older than 50 years. These results are in line with other previous research works on the general efficacy of vaccines [27] and point to a better capacity and higher activity of the immunological system in younger people in the detection of potentially noxious pathogens as the main reason for this. Thus, the immunological response of the organism decreases with age, which in turn causes a lower immunological reaction against vaccines. Indeed, senectitude is a risk factor that predisposes one to contagious infection due to a “physiological immunodepression”, which supports the data found on the lower efficacy of vaccines in this population group [28].

Other research studies at a Spanish level have found differences in the efficacy of hepatitis B vaccines according to gender, with lower efficacy in male healthcare providers [29]. While our article could not replicate these results, since healthcare workers in our health center are women by a large majority, there is a similar number of male and female workers in our study—11 male and 15 female workers—which could be an indication of a higher proportion of nonresponsive male workers to Engerix B^®^ vaccination. Again, this limitation in the number of study subjects may have prevented us from replicating the results found by the aforementioned work.

Within the limitations of this research, sample size must be again mentioned. With a higher number of test subjects, some or all of the previously discussed correlations found in other research works might have been reproduced. Increasing sample size would hence be the logical solution to this issue; however, the requirements and specifics necessary to be considered as a candidate to participate in this research make it difficult to include a higher number of test subjects at this moment. In the future, a collaboration with other health centers at the national level could help overcome this limitation. Another limitation was that the study does not have a control arm with additional doses of Engerix B^®^ or similar vaccine to compare with the results of Fendrix^®^.

The findings discussed herein could support the argument for the inclusion of higher efficacy vaccines such as Fendrix^®^ for healthcare workers exposed to the HBV pathogen and who were nonresponsive to the regular, standardized vaccination schedule. Several research groups explain the possibility of extending the standardized use of highly effective vaccines, such as Fendrix^®^ or HBVAXPRO^®^, for this type of target population who are highly exposed to contact with HBV, offering them protection and a lot of advantages to the society [30]. Increasing the immunity against this virus amongst the target population could reduce the risk faced regarding future work-related biological accidents. This would result in both reduced negative health effects for health personnel and a reduced economic cost since the target population is small. This would result in a positive outcome regarding socio-economic costs in the healthcare system and the psychological health of healthcare workers.

## 5. Conclusions

The use of adjuvanted vaccines such as Fendrix^®^ allows the development of immunity against pathogens, such as hepatitis B virus, against which standard vaccines have not resulted in serological protection for the inoculated patients. With its inclusion of said vaccine on the standard immunization schedule of nonresponsive healthcare workers, higher protection for this population group could be provided against hepatitis B, as they are exposed to constant risk of contagions with these pathogens in the form of injuries with contaminated sharp medical instrumentation and splatter from bodily fluids.

## Figures and Tables

**Table 1:** Correlation between immunity, age, and antibody titer (*n* = 26).

	Immunity		
Variable	Yes	No	*p*
Age (years) *	45.58 ± 8.19	52.50 ± 0.71	0.032
anti-HBs ** (mIU/mL)	351.83 ± 302.14	3.10 ± 4.38	0.021

Bivariate analysis of the relationship between immunity with the other variables using Student’s *t*-distribution * and Mann–Whitney U test **.

**Table 2:** Correlation between gender and anti-HBs titer with the remaining variables (*n* = 26).

Variable		Gender		*p*
		Male	Female	
Age (years) *		44.73 ± 9.06	47.13 ± 7.43	0.464
No. of doses **		2.18 ± 1.08	2.00 ± 1.31	0.646
anti-HBs ** (mIU/mL)		435.04 ± 324.45	244.31 ± 272.64	0.164
Immunity ***	**Yes**	11	13	0.207
	**No**	0	2	
**Variable**		**N. Doses**		***p***
anti-HBs **** (mIU/mL)		−0.347		0.083

Bivariate analysis of the relationship between immunity with the other variables using Student’s *t*-distribution *, Mann–Whitney U test **, chi-squared test (χ^2^) *** and Rho-Spearman ****.

## Data Availability

The data presented in this study are available on request from the corresponding author.
